# Pain Response and Associated Factors During Bacillus Calmette-Guérin Vaccination in Newborns: A Cross-Sectional Study

**DOI:** 10.7759/cureus.109290

**Published:** 2026-05-20

**Authors:** Chau Vu Bao Nguyen, Trang Phan Thao Vo, Mi Ha Vu, Nguyet Vu Minh Nguyen, Nguyen Thi Hong Dang, Trang Thi Kim Nguyen, Linh Tran Phuong Giang, Thu-Tinh Nguyen

**Affiliations:** 1 Department of Neonatology, University Medical Center Ho Chi Minh City, Ho Chi Minh City, VNM; 2 Department of Public Health, Pham Ngoc Thach University of Medicine, Ho Chi Minh City, VNM; 3 Department of Pediatrics, School of Medicine, University of Medicine and Pharmacy at Ho Chi Minh City, Ho Chi Minh City, VNM; 4 Neonatal Intensive Care Unit, Children's Hospital 2, Ho Chi Minh City, VNM

**Keywords:** bcg vaccine, immunization, newborn, nips scale, pain score

## Abstract

Background: Bacillus Calmette-Guérin (BCG) vaccination is routinely administered shortly after birth in many countries, including Vietnam. Although brief, this procedure may induce substantial pain during a critical period of neonatal neurodevelopment. Evidence on pain responses during BCG vaccination and modifiable procedural factors remains limited. This study aimed to assess neonatal pain during BCG vaccination and identify factors associated with severe pain.

Methods: We conducted a cross-sectional observational study involving 172 newborns aged less than 48 hours who received intradermal BCG vaccination at a tertiary hospital in Ho Chi Minh City, Vietnam. Pain was assessed immediately before vaccination and immediately after needle withdrawal using the Neonatal Infant Pain Scale (NIPS). Severe pain was defined as a NIPS score of 5 or higher. Infant characteristics and procedural variables, including sex, mode of delivery, infant feeding prior to vaccination, and vaccination order (BCG first vs. BCG injected after hepatitis B vaccination), were recorded. Associations with severe pain were examined using Poisson regression with robust variance.

Results: The mean NIPS score after BCG vaccination was 3.83 ± 1.72. Overall, 64/172 newborns (37.2%) experienced severe pain (NIPS score ≥ 5). Feeding before vaccination was associated with a lower prevalence of severe pain (aPR = 0.35; 95% CI: 0.19-0.65), whereas receiving BCG after hepatitis B vaccination (aPR = 1.87; 95% CI: 1.27-2.77) and assisted delivery (aPR = 1.93; 95% CI: 1.01-3.66) were associated with a higher prevalence of severe pain.

Conclusion: Procedural pain during BCG vaccination was common among newborns. Feeding before vaccination, vaccination order, and assisted delivery were associated with neonatal pain response and may represent clinically relevant procedural factors. Given the single-center observational design, these findings should be interpreted cautiously and require confirmation in a controlled intervention. Simple, feasible pain-reduction strategies should be considered during routine neonatal vaccination.

## Introduction

Tuberculosis (TB) remains a major global public health problem and continues to impose a substantial disease burden in Vietnam. According to the World Health Organization (WHO), Vietnam was among the 30 highest TB-burden countries worldwide in 2023, ranking 11th globally. An estimated 172,000 new TB cases and 13,000 TB-related deaths occur annually in Vietnam, excluding multidrug-resistant TB [1]. TB control remains challenging due to persistent transmission, dense populations, and socioeconomic disparities, especially among vulnerable groups such as young children and neonates. Bacillus Calmette-Guérin (BCG) vaccination is the cornerstone of TB prevention in early life and has been included in Vietnam’s Expanded Program on Immunization since 1985. With consistently high coverage exceeding 95%, BCG vaccination is estimated to be 70-80% effective in preventing severe forms of childhood TB, such as tuberculous meningitis and miliary TB, thereby contributing substantially to reductions in TB-related mortality among young children [2]. Early administration of BCG after birth is recommended to provide prompt protection during the period of highest susceptibility to disseminated TB [3].

Despite its proven benefits, BCG vaccination is an invasive procedure that causes acute pain in newborns. Although brief, this pain occurs during a critical period of neurological development. Evidence from previous studies suggests that inadequately managed pain in early life may be associated with altered pain processing and adverse neurodevelopmental outcomes later in childhood [4,5]. Neonatal procedural pain may also affect parents and healthcare providers, potentially contributing to psychological distress and vaccine hesitancy [6]. Multiple variables influence neonatal pain response, such as sex, gestational age, delivery method, and other clinical factors. Consequently, effective pain management during neonatal vaccination is considered both an ethical and clinical priority. Several non-pharmacological interventions, including pacifier use, oral glucose, and breastfeeding, have been used to reduce neonatal pain, with feeding before vaccination described as a simple and cost-effective approach [7-9]. These interventions are particularly relevant in resource-limited settings, where pharmacological analgesia may not be routinely available or feasible [10]. Objective assessment of neonatal pain requires standardized and validated measurement tools. The Neonatal Infant Pain Scale (NIPS) is a widely used behavioral pain assessment instrument that evaluates pain responses using six observable indicators. The NIPS has been translated into Vietnamese and validated for clinical use, supporting its applicability in the local healthcare setting [9,11]. Although BCG vaccination is routinely administered in Vietnam, quantitative data on pain intensity and associated procedural factors remain limited. Therefore, this study aimed to assess neonatal pain scores during BCG vaccination using the NIPS and to identify factors associated with severe pain.

## Materials and methods

Ethics approval

The study protocol was reviewed and approved by the Institutional Ethics Committee for Biomedical Research, University Medical Center Ho Chi Minh City, Vietnam (Approval No. 154/GCN-HĐĐĐ, dated October 10, 2025). Written informed consent was obtained from the parents or legal guardians of all participating newborns prior to enrollment. Participant confidentiality and data anonymity were strictly maintained throughout the study.

Study design and setting

A cross-sectional observational study was conducted to assess neonatal pain responses before and after intradermal BCG vaccination at the Vaccination Unit of University Medical Center Ho Chi Minh City. This center is a tertiary-level public hospital operating under a university-hospital model and staffed by specialized medical professionals. The hospital has approximately 1,000 inpatient beds and provides an estimated 7000-10000 outpatient visits per day, serving as a major referral center for advanced medical care in Southern Vietnam. Data collection was carried out over a one-month period, from October 2025 to November 2025.

Participants

Inclusion Criteria

The study population consisted of healthy newborns receiving their first dose of the BCG vaccine. Eligible participants were clinically stable newborns aged less than 48 hours at the time of vaccination, born at University Medical Center Ho Chi Minh City, with a gestational age of at least 34 weeks, and presented for their first BCG vaccination at the vaccination unit of the Department of Neonatology at the same institution. Clinically stable newborns were defined as infants without respiratory distress, hemodynamic instability, or need for intensive care support at the time of vaccination. Written informed consent was obtained from parents or legal guardians prior to enrollment.

Exclusion Criteria

Newborns were excluded if they had underlying medical conditions, including postnatal respiratory distress, neonatal infection, or multiple congenital anomalies.

Study size

The sample size was calculated primarily for estimating the mean post-vaccination NIPS score. Analyses of factors associated with severe pain were considered exploratory.

The required sample size for estimating a population mean was calculated using the formula:

$n \ge \left(\frac{Z_{1-\alpha/2}\sigma}{\delta}\right)^2$

Where:

Z1−α/2​=1.96, corresponding to a two-sided significance level of 0.05.

σ=2.5 represents the assumed standard deviation, derived from the study by Im et al. [12].

δ=0.4 denotes the desired margin of error for the mean NIPS score.

Substituting these parameters into the formula yields:

$n \ge \left( \frac{1.96 \times 2.5}{0.4} \right)^2 = 150.06$

Consequently, a minimum sample size of 151 newborns was required.

Variables

The primary dependent outcome measure was neonatal pain score assessed immediately before BCG vaccination and immediately after needle withdrawal, using the Neonatal Infant Pain Scale (NIPS). Subsequently, pain scores were categorized into two groups: severe pain (defined as NIPS score ≥ 5) and non-severe pain (NIPS score < 5) [9,11]. Independent variables included sex, mode of delivery, vaccination order (BCG first vs. BCG after hepatitis B vaccination), and feeding before vaccination, defined as initiation of the most recent feeding within 45 minutes before BCG vaccination.

Study protocol

All data collection was conducted from October 2025 to November 2025 by a single trained nurse who received standardized training in pain assessment using the Neonatal Infant Pain Scale (NIPS). Eligible newborns were identified consecutively from the daily vaccination list. All newborns meeting the inclusion criteria were screened, and the numbers screened and excluded, with reasons for exclusion, were recorded.

Written informed consent was obtained from parents or legal guardians. During vaccination, infants were held by a family member, with clothing adjusted to allow observation of breathing patterns. Limb immobilization was applied only at the time of vaccination. According to routine practice, newborns received both BCG and hepatitis B vaccines on the same day, administered by the same healthcare worker. The order of vaccination followed routine practice and was recorded.

Pain was assessed using the NIPS immediately before BCG vaccination and immediately after needle withdrawal, following vaccine administration. The NIPS is a validated behavioral pain assessment tool with a total score ranging from 0 to 7, derived from six observable indicators: facial expression, crying, breathing pattern, arm movement, leg movement, and state of arousal. The total scores of 0 to 2 were considered no or mild pain, scores of 3 to 4 indicated moderate pain, and scores of 5 to 7 indicated severe pain [9,11] (Table 1).

**Table 1:** Components and scoring criteria of the Neonatal Infant Pain Scale (NIPS)

Parameter	Finding	Points
Facial expression	Relaxed	0
	Grimace	1
Cry	No cry	0
	Whimper	1
	Vigorous crying	2
Breathing patterns	Relaxed	0
	Change in breathing	1
Arms	Restrained	0
	Relaxed	0
	Flexed	1
	Extended	1
Legs	Restrained	0
	Relaxed	0
	Flexed	1
	Extended	1
State of arousal	Sleeping	0
	Awake	0
	Fussy	1

Before vaccination, infants were required to be calm and non-stimulated, defined by a baseline NIPS score of 0-1. If the hepatitis B vaccine was administered before BCG, BCG vaccination was delayed until the infant stabilized and the NIPS score returned to baseline. Feeding before vaccination was defined as initiation of the most recent feeding within 45 minutes before BCG vaccination, based on prior clinical studies suggesting that recent feeding may provide a clinically relevant analgesic effect during neonatal procedures [13]. All pain assessments were performed and recorded by the trained nurse.

Statistical analysis

Data were entered using EpiData (version 3.1, EpiData Association, Odense, Denmark, 2008) and analyzed using Stata (version 17, StataCorp LLC, College Station, TX, USA, 2021). Descriptive statistics were used to summarize participant characteristics. Categorical variables were reported as frequencies and percentages, and continuous variables as mean ± standard deviation or median (interquartile range). Mean post-vaccination NIPS scores between vaccination-order groups were compared using the independent-samples t-test.

A multivariable Poisson regression model with robust variance was used to evaluate the association between independent factors and severe pain (defined as a NIPS score of 5 or more) during BCG vaccination. Variables were selected a priori based on clinical relevance and prior literature. Associations were quantified using prevalence ratios (aPRs) with 95% confidence intervals.

## Results

**Figure 1:**
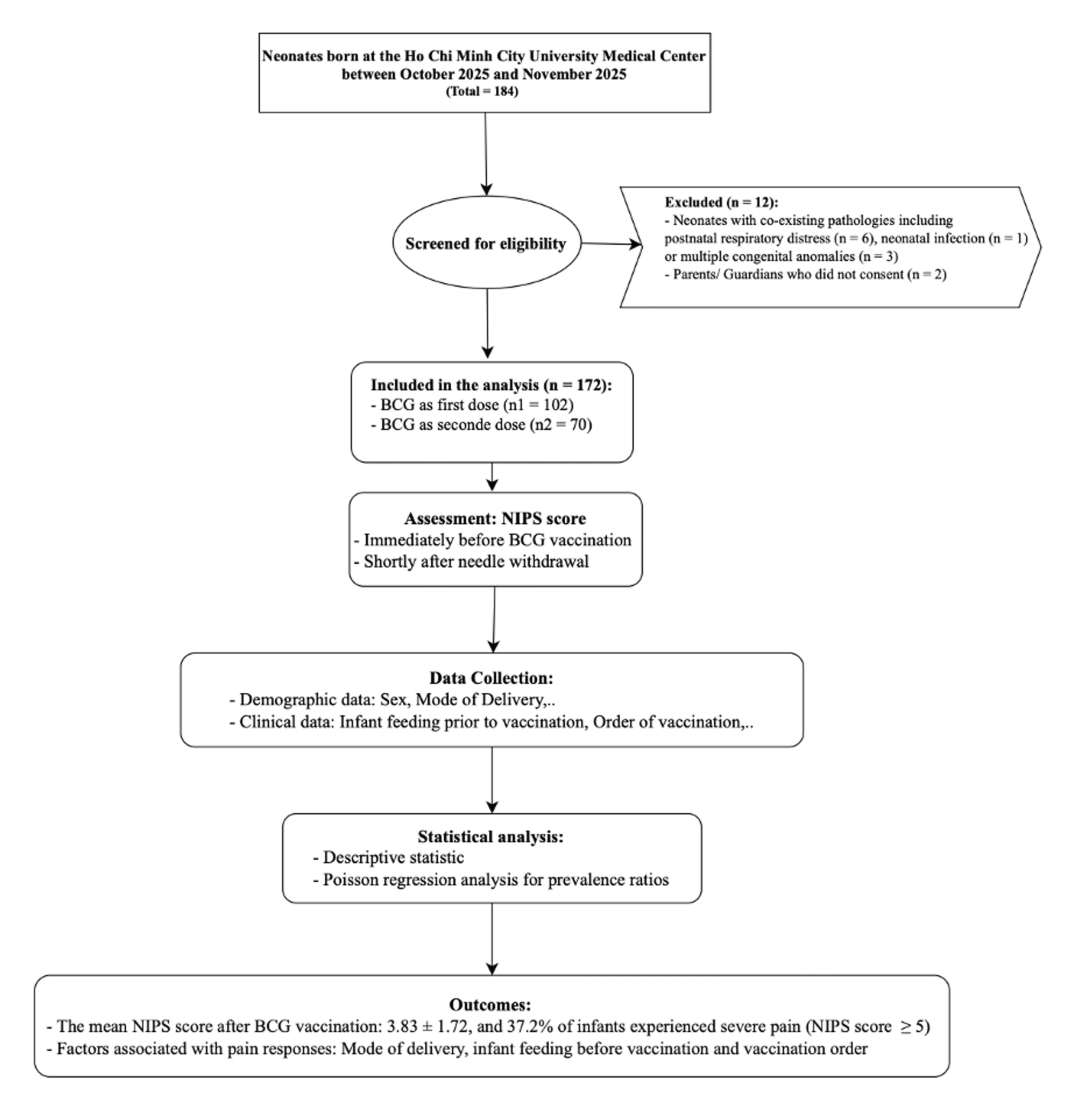
Flowchart of participant recruitment, pain assessment, and analysis Flowchart of participant screening, exclusion, pain assessment, data collection, and analysis. Of 184 neonates screened, 12 were excluded, and 172 were included in the study. Pain response was assessed before BCG vaccination and shortly after needle withdrawal using the NIPS. BCG: Bacillus Calmette-Guérin; NIPS: Neonatal Infant Pain Scale; PR: prevalence ratio

From October to November 2025, a total of 172 newborns met the inclusion criteria and were enrolled in the study. No enrolled cases were excluded, and complete data were available for all 172 participants (Figure 1).

Of these, males accounted for 57.0% (n = 98) and females for 43.0% (n = 74). Mean birth weight was 3057 ± 401 grams, and mean gestational age was 38.4 ± 1.3 weeks. Regarding mode of delivery, cesarean section accounted for 55.8% (n = 96), vaginal delivery for 39.5% (n = 68), and assisted delivery for 4.7% (n = 8). Regarding vaccination order, BCG was injected first in 102 newborns (59.3%) and second, after hepatitis B vaccination, in 70 newborns (40.7%) (Table 2).

**Table 2:** Baseline characteristics of the study sample (N = 172) Data are presented as n (%) or mean ± SD (range). BCG: Bacillus Calmette-Guérin

Characteristic	Value
Gestational age (weeks)	38.4 ± 1.3 (34–40)
Birth weight (grams)	3057 ± 401 (1850–4100)
Sex	
Male	98 (57.0)
Female	74 (43.0)
Mode of delivery	
Vaginal delivery	68 (39.5)
Cesarean section	96 (55.8)
Assisted delivery	8 (4.7)
Vaccination order	
BCG first	102 (59.3)
BCG second (after hepatitis B vaccination)	70 (40.7)
Time since last feeding before vaccination (minutes)	84.5 ± 55.2 (10–240)

The mean post-vaccination NIPS score was 3.83 ± 1.72, with observed scores ranging from 0 to 7. Based on the Neonatal Infant Pain Scale (NIPS) classification, 64 infants (37.2%) were categorized as having severe pain. Newborns who received BCG after hepatitis B had higher post-vaccination mean NIPS scores than those who received BCG first (4.40 ± 1.61 vs. 3.43 ± 1.69; p < 0.001) (Table 3).

**Table 3:** Pain scores before and after BCG vaccination (N=172) Pain scores are presented as n (%) or mean ± SD (range). BCG: Bacillus Calmette-Guérin

Measure	Value
Before vaccination	
0–1, n (%)	172 (100)
>1, n (%)	0 (0)
After vaccination, overall	
Non-severe pain (0–4), n (%)	108 (62.8)
Severe pain (5–7), n (%)	64 (37.2)
Post-vaccination pain score, mean ± SD (range)	3.83 ± 1.72 (0–7)
After vaccination, by vaccination order, mean ± SD (range)	
BCG first (n = 102)	3.43 ± 1.69 (0–7)
BCG second (after hepatitis B vaccination) (n = 70)	4.40 ± 1.61 (0–7)

In multivariable Poisson regression with robust variance, feeding before vaccination was associated with a lower prevalence of severe pain (aPR = 0.35; 95% CI: 0.19-0.65; p = 0.001), whereas BCG injection after hepatitis B vaccination (aPR = 1.87; 95% CI: 1.27-2.77; p = 0.002) and assisted delivery (aPR = 1.93; 95% CI: 1.02-3.66; p = 0.043)were associated with a higher prevalence of severe pain (Table 4).

**Table 4:** Multivariable analysis of factors associated with severe pain during BCG vaccination (N=172) BCG: Bacillus Calmette-Guérin; aPR, adjusted prevalence ratio; CI, confidence interval; Ref, reference group Data are presented as n (%). Percentages are row percentages. * Adjusted prevalence ratios (aPRs) and 95% confidence intervals were estimated using a multivariable Poisson regression model with robust variance. The model included sex, mode of delivery, vaccination order, and infant feeding before vaccination, with severe pain (NIPS score ≥ 5) as the outcome. Gestational age and birth weight were not included because they were not predefined variables in the present analysis. $ “Feeding before vaccination” was defined as initiation of the most recent feeding within 45 minutes before BCG vaccination, based on prior evidence supporting a clinically relevant analgesic window for recent feeding during neonatal procedures.

Variable	Category	Severe pain, n/N (%)	aPR (95% CI)*	p-value
Sex	Male	39/98 (39.8)	Ref	-
	Female	25/74 (33.8)	0.85 (0.57–1.27)	0.425
Mode of delivery	Vaginal delivery	22/68 (32.3)	Ref	-
	Cesarean section	37/96 (38.5)	1.19 (0.77–1.82)	0.423
	Assisted delivery	5/8 (62.5)	1.93 (1.01–3.66)	0.043
Vaccination order	BCG first	28/102 (27.5)	Ref	-
	BCG second (after hepatitis B vaccination)	36/70 (51.4)	1.87 (1.27–2.77)	0.002
Feeding before vaccination^$^	No	55/117 (47.0)	Ref	-
	Yes	9/55 (16.4)	0.35 (0.19–0.65)	0.001

## Discussion

This study showed that BCG vaccination was frequently associated with clinically meaningful pain in newborns and identified procedural factors associated with severe pain response. Because of the cross-sectional observational design, these findings should be interpreted as associations rather than causal effects. Baseline characteristics of the study population were broadly comparable with national reports, although the cesarean section rate appeared higher in our study setting [14]. This higher proportion may reflect referral patterns or institutional practices typical of tertiary care facilities.

The mean pain score following BCG vaccination was 3.83 ± 1.72, with more than one-third of newborns experiencing severe pain. These findings are consistent with previous reports and highlight that pain associated with routine neonatal vaccination is substantial and warrants greater clinical attention. Given the documented association between early-life pain and neurodevelopmental and behavioral outcomes [4,5], effective pain-reduction strategies during neonatal procedures are important. Pain management during neonatal BCG vaccination remains underemphasized, particularly in resource-limited settings. In clinical practice, this issue often receives limited attention, and supportive measures are not routinely implemented. This gap between evidence and practice may be related to workload constraints, limited training in neonatal pain assessment, and the perception that vaccination-related pain is unavoidable. Several interventions have been reported to reduce pain scores and crying duration following immunization, including swaddling, skin-to-skin contact, and feeding during or prior to injection [7,10,15,16], oral sucrose administration [8], and the use of 5% lidocaine-prilocaine cream [5]. Among these approaches, non-pharmacological interventions are particularly attractive because they are safe, inexpensive, and can be readily integrated into routine workflows without requiring additional equipment. Based on prior evidence from clinical trials, feeding within 45 minutes before BCG vaccination was selected for subsequent analyses, reflecting a clinically relevant time window that may help reduce neonatal procedural pain [13].

Among the factors associated with severe pain, mode of delivery, infant feeding prior to vaccination, and vaccination order were statistically significant. Of these, feeding before vaccination represents a potentially modifiable factor that can be readily implemented in clinical practice. Identifying modifiable procedural factors is important because they may provide actionable opportunities to improve neonatal care without major structural changes. Assisted delivery was associated with increased pain risk; however, this factor depends on obstetric indications and is not easily modifiable. This result should be interpreted cautiously due to the small number of assisted deliveries, which may limit the precision and stability of the estimate. The vaccination order was associated with pain response [17]. Newborns who received BCG after hepatitis B vaccination had higher post-vaccination pain scores than those who received BCG first. This finding may reflect the effect of recent prior painful exposure, but causality cannot be inferred from the present study. At present, there are no national Vietnamese guidelines specifying the order of BCG and hepatitis B vaccine administration in newborns; in routine clinical practice, the vaccination sequence is generally determined by local workflow patterns and healthcare workers’ preferences or habits. These findings suggest that vaccination order may influence cumulative procedural pain; however, this hypothesis should be tested in controlled trials before recommendations are made [17,18]. Future randomized studies comparing vaccination sequences could help determine whether modifying injection order reduces overall procedural pain without compromising immunization efficiency. Feeding prior to vaccination was associated with a lower prevalence of severe pain, consistent with previous evidence supporting the analgesic effects of breastfeeding or recent feeding during neonatal procedures [7,10]. As a simple, low-cost, and non-invasive intervention, encouraging feeding before vaccination may be a feasible approach to improve neonatal comfort in routine clinical settings.

Limitations

The findings of this study are most applicable to tertiary-level hospitals with similar patient populations and clinical workflows. Generalizability to primary healthcare facilities may be limited, and multicenter studies across different healthcare levels are needed to confirm these associations. Several limitations should be considered. First, pain assessment was performed by a single trained observer, and blinding was not feasible, introducing potential observer bias. Although a single assessor may improve scoring consistency, it precluded assessment of inter-rater reliability. Second, pain evaluation relied solely on the NIPS behavioral scale without complementary physiological measures, such as heart rate or oxygen saturation, which may limit the comprehensiveness of pain evaluation. Third, potentially relevant confounders, including gestational age, birth weight, prior painful exposure, and environmental factors during vaccination, were not included in the multivariable model. Fourth, the small number of newborns in certain subgroups, particularly assisted delivery, may limit the precision of the corresponding estimates. Finally, the cross-sectional observational design limits causal inference.

Routine neonatal procedures such as vaccination can produce clinically meaningful pain, and early-life pain exposure may have lasting consequences [10,15,19]. These findings emphasize the importance of integrating simple, evidence-based pain-reduction strategies into neonatal vaccination practices. Further high-quality studies are needed to support the implementation of standardized pain management protocols in neonatal care.

## Conclusions

This study found that a substantial proportion of newborns experienced severe pain during BCG vaccination, as assessed by the NIPS scale. Feeding before vaccination was associated with a lower prevalence of severe pain, whereas receiving BCG after hepatitis B vaccination and assisted delivery were associated with higher pain scores. Because of the single-center observational design, these associations should not be interpreted as causal and may not be fully generalizable to other healthcare settings. Nevertheless, the findings support consideration of simple, feasible pain-reduction strategies during routine neonatal vaccination. Further large-scale interventional studies, including randomized controlled trials, are required to confirm these associations and to inform evidence-based clinical recommendations for neonatal pain management during vaccination.
